# Dietary mastic oil extracted from *Pistacia lentiscus* var. *chia* suppresses tumor growth in experimental colon cancer models

**DOI:** 10.1038/s41598-017-03971-8

**Published:** 2017-06-19

**Authors:** Katerina Spyridopoulou, Angeliki Tiptiri-Kourpeti, Evangeli Lampri, Eleni Fitsiou, Stavros Vasileiadis, Manolis Vamvakias, Haido Bardouki, Anna Goussia, Vasiliki Malamou-Mitsi, Mihalis I. Panayiotidis, Alex Galanis, Aglaia Pappa, Katerina Chlichlia

**Affiliations:** 1 Department of Molecular Biology and Genetics, Democritus University of Thrace, University Campus-Dragana, Alexandroupolis, 68100 Greece; 2 Department of Pathology, School of Health Sciences, University of Ioannina, University Campus, Ioannina, 45110 Greece; 3 VIORYL S.A., 28th km National Road Athens - Lamia, Afidnes, 19014 Greece; 4 Department of Applied Sciences, Faculty of Health & Life Sciences, Northumbria University, Ellison Building A516, Newcastle Upon Tyne, NE1 8ST United Kingdom

## Abstract

Plant-derived bioactive compounds attract considerable interest as potential chemopreventive anticancer agents. We analyzed the volatile dietary phytochemicals (terpenes) present in mastic oil extracted from the resin of *Pistacia lentiscus* var. *chia* and comparatively investigated their effects on colon carcinoma proliferation, a) *in vitro* against colon cancer cell lines and b) *in vivo* on tumor growth in mice following oral administration. Mastic oil inhibited - more effectively than its major constituents- proliferation of colon cancer cells *in vitro*, attenuated migration and downregulated transcriptional expression of survivin (BIRC5a). When administered orally, mastic oil inhibited the growth of colon carcinoma tumors in mice. A reduced expression of Ki-67 and survivin in tumor tissues accompanied the observed effects. Notably, only mastic oil -which is comprised of 67.7% α-pinene and 18.8% myrcene- induced a statistically significant anti-tumor effect in mice but not α-pinene, myrcene or a combination thereof. Thus, mastic oil, as a combination of terpenes, exerts growth inhibitory effects against colon carcinoma, suggesting a nutraceutical potential in the fight against colon cancer. To our knowledge, this is the first report showing that orally administered mastic oil induces tumor-suppressing effects against experimental colon cancer.

## Introduction

Plant-derived bioactive compounds attract nowadays considerable interest as potential chemopreventive anticancer agents. Chemoprevention refers to the strategy of using bioactive natural or synthetic compounds to inhibit cancer progress^1^. Naturally occurring compounds in plants, phytochemicals, have been under thorough investigation for the identification of potent anti-cancer agents with great success and, thus, are considered the backbone of pharmaceutical innovation^2^. Dietary phytochemicals which derive from edible plants such as vegetables, fruits and herbs, form a distinct and very promising class of chemopreventive nutraceuticals. In particular, certain bioactive dietary phytochemicals (e.g. curcumin) have been shown to contribute to colon cancer prevention or therapy^3, 4^.

The incidence of colorectal cancer is now increasing in countries where it was previously low, such as certain Asian (e.g. Japan) and Eastern European (e.g. Czech Republic) countries^5^, indicating the influence of westernized lifestyle and specifically unhealthy diet on the prevalence of colon cancer risk. It has been estimated that a substantial proportion (approximately 35%) of new cancer cases in Western countries, can be prevented only by dietary means^6^, suggesting that dietary modifications or nutritional interventions may be beneficial for colorectal cancer prevention.

Essential oils from aromatic plants have been shown to possess diverse biological activities^7^ and are a great source of dietary phytochemicals, being mixtures of biologically highly active compounds^8^. One of the main classes of chemical compositions found in essential oils are isoprenic derivatives, that include monoterpenes and sesquiterpenes^9^. Terpenes (mono and sesquiterpenes) are the most prevalent constituents in essential oils. Monoterpenes, consist of two isoprenic units (C_10_) and can be linear or cyclic, allowing for a great structural diversity^9^. A number of these monoterpenes such as limonene and perillic acid have been reported to possess antitumor activity in rodent models^10^.

Mastic oil (MO) is the essential oil extracted from the resin (mastic gum) of the plant *Pistacia lentiscus* var. *chia*, a plant that has been cultivated for its aromatic resin mostly in the southern part of Chios island in Greece. MO is a dietary plant extract that, apart from being traditionally used as food additive and flavoring agent, has also been incorporated in folk medicine of various ethnic groups for the treatment of gastrointestinal disorders. Chemical analysis showed that MO mainly consists of volatile terpenes. Two aromatic monoterpenes, α-pinene and myrcene, have been identified as its major constituents^11^. The monoterpene α-pinene exhibited antimetastatic effects when administered intraperitoneally in a mouse melanoma model^12^ and suppressed hepatocellular carcinoma growth in a mouse xenograft model^13^, while myrcene exhibited analgesic^14^ and anti-inflammatory^15^ activity.

During the past decade, there has been a growing literature on the anticancer potential of extracts derived from mastic resin^16^. The resin was shown to suppress proliferation of prostate^17^ and hepatic cancer cells *in vitro*, as well as, leukemia, oral squamous and glioblastoma cell lines^18^. Moreover, MO was proven to inhibit efficiently the growth of leukemia cells *in vitro* ^19^, and Lewis lung carcinoma cells both *in vitro* and *in vivo*, when administered intraperitoneally in a Lewis lung adenocarcinoma model in syngeneic mice^20, 21^. Additionally, resin’s both hexane and ethanolic extracts were found to inhibit HCT116 colon cancer cell proliferation *in vitro* ^22, 23^, while intraperitoneal administration of the hexane extract attenuated growth of HCT116 colorectal tumors xenografted into SCID mice^24^.

Although antiproliferative activities against cancer cell lines have been reported for *Chios* mastic, phytochemical constituents in MO have not been comparatively analyzed for their antiproliferative effects *in vitro* and *in vivo*, and likewise, *Chios* mastic gum has not been extensively studied against colon cancer. Moreover, there is evidence for potential anti-tumor activity of MO. However, despite its traditional use as food additive, little information is available on its activity against colon cancer, and no information on the antiproliferative potential of orally administered MO in experimental tumor models. Moreover, MO’s major monoterpenes have not yet been extensively studied. The aim of this study was to investigate potential antiproliferative effects of the individual monoterpenes present in MO as well as MO itself (as a combination/mixture of its constituents). In addition, we assessed whether oral administration of MO has antitumor potential against colon cancer and, if so, to what extent its anti-tumor activity can be attributed to its main constituents.

Therefore, in the present study we analyzed the dietary volatile phytochemicals present in mastic essential oil extracted from the resin of *Pistacia lentiscus* var. *chia* and investigated the effects of MO and its most prevalent monoterpenes on colon carcinoma proliferation, a) *in vitro* in colon cancer cell lines and b) *in vivo* on tumor growth in mice following oral administration.

## Results

### Extraction of mastic oil and GC/MS analysis of its volatile constituents

**Figure 1:**
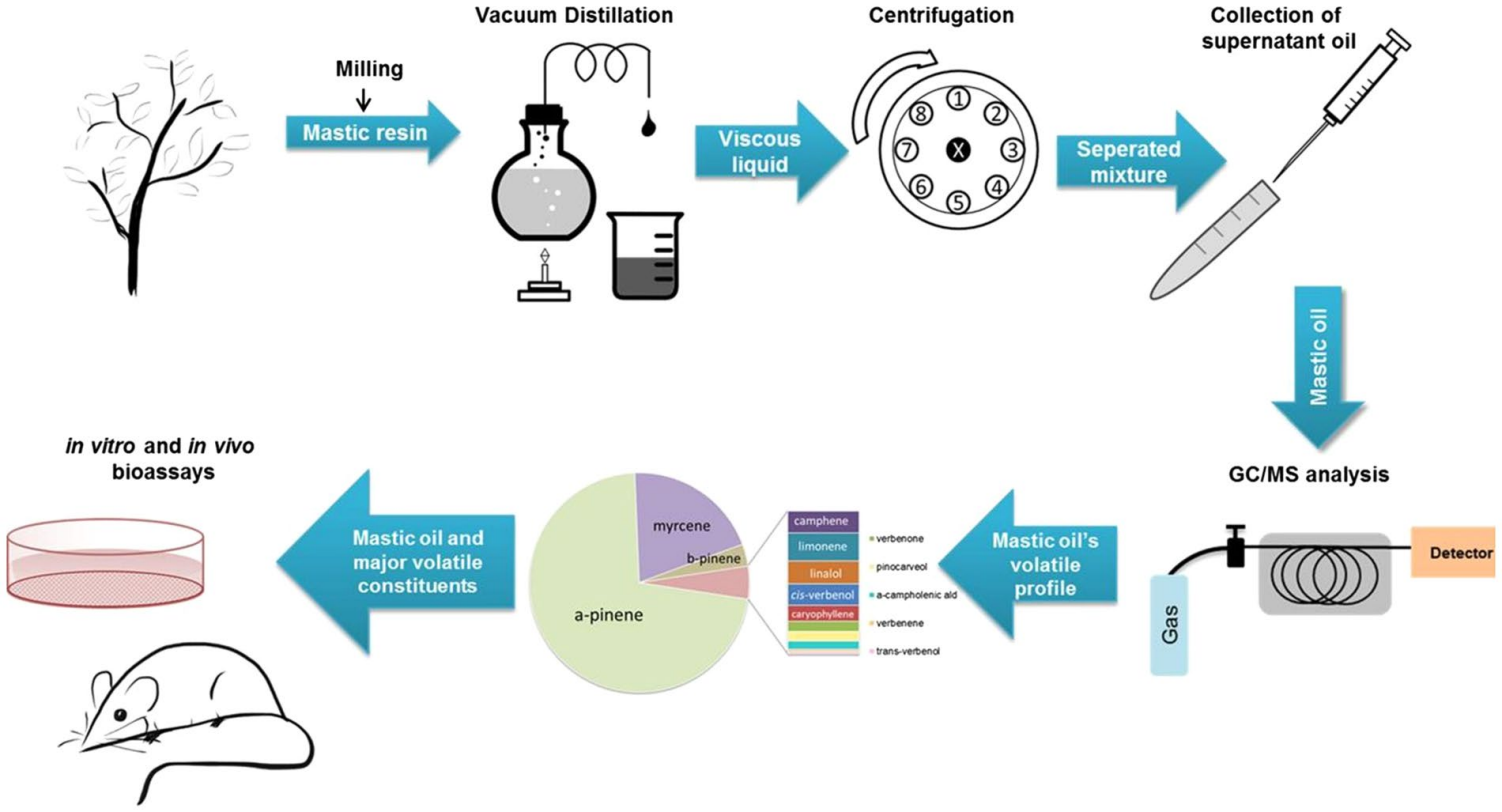
Schematic representation of the MO extraction procedure and analysis of its constituents. MO was extracted from the resin of the plant *Pistacia lentiscus* var. *chia* through vacuum distillation and its volatile profile was analyzed by GC/MS. MO and its identified major constituents were comparatively tested for their potential anticancer properties *in vitro* and *in vivo*.

**Figure 2:**
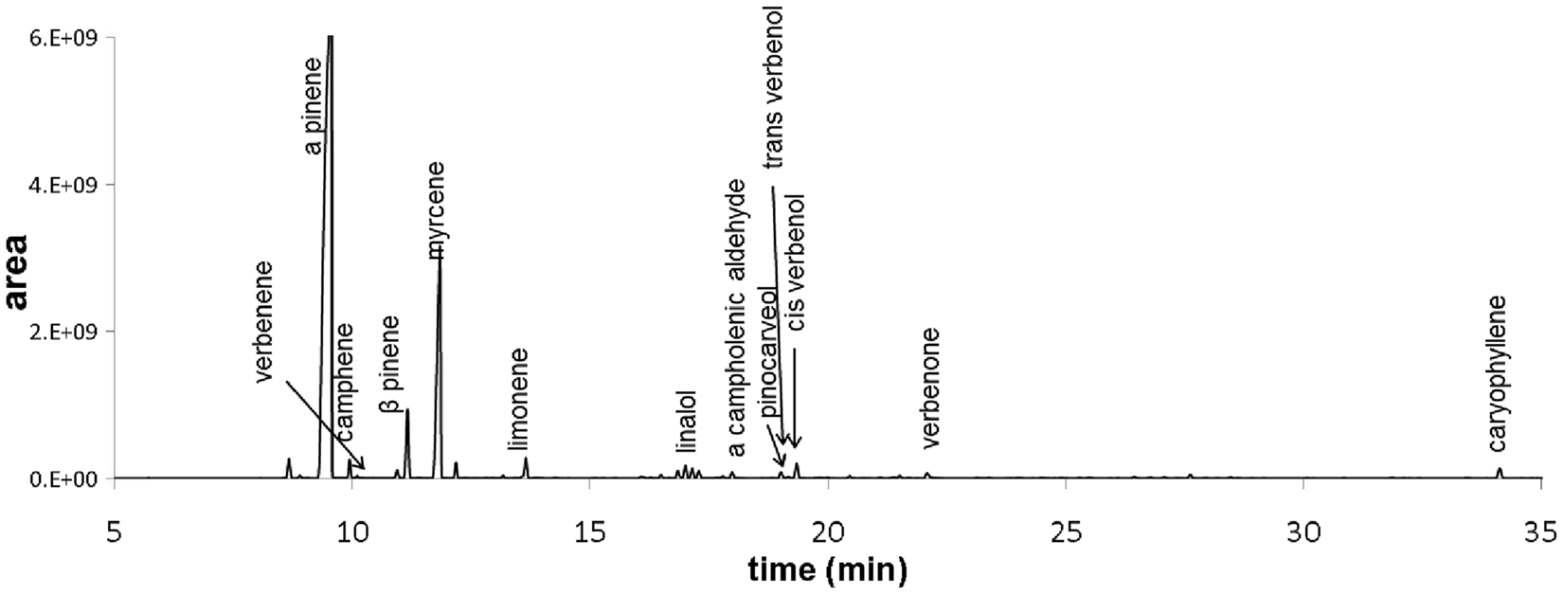
Gas chromatogram of extracted MO. Analysis of volatile compounds in mastic oil was performed by the capillary GC-MS on an Agilent mass selective detector system. Compound identification (labeled signals) was based on a comparison of the retention indices and mass spectra with those of authentic samples.

MO was extracted from the resin of the plant *Pistacia lentiscus* var. *chia* by distillation (Fig. 1). The total distillate was used. The resin, also known as “mastic gum”, was provided by Chios Mastic Gum Growers Association L.L.C. (Chios, Greece). Volatile profile analysis by GC/MS identified the composition of MO (Fig. 2). MO can be considered as a mixture of individual phytochemicals. In particular, volatile monoterpenes and a sesquiterpene (caryoplyllene) were identified, present at different percentages (Table 1), and covering 94.12% of the total chromatographic area. MO and the 5 most abundant monoterpenes, α-pinene (67.71%), myrcene (18.81%), β-pinene (3.05%), limonene (0.89%) and linalol (0.73%), were further analyzed for their antiproliferative activity.

**Table 1:** Volatile compounds present in MO documented by GC-MS analysis.

KRI^*^	compounds	relative (%) area	structure	formula	MW^**^
920	***α*** **-pinene**	67.71		C_10_H_16_	136.24
934	camphene	0.70		C_10_H_16_	136.24
937	verbenene	0.07		C_10_H_14_	134.22
958	***β*** **-pinene**	3.05		C_10_H_16_	136.24
976	**myrcene**	18.81		C_10_H_16_	136.24
1010	**limonene**	0.89		C_10_H_16_	136.24
1086	**linalol**	0.73		C_10_H_18_O	154.25
1094	*α*-campholenic ald	0.26		C_10_H_16_O	152.23
1113	pinocarveol	0.32		C_10_H_16_O	152.23
1117	*trans*-verbenol	0.07		C_10_H_16_O	152.23
1120	*cis*-verbenol	0.69		C_10_H_16_O	152.23
1168	verbenone	0.32		C_10_H_14_O	150.22
1405	caryophyllene	0.50		C_15_H_24_	204.36

*KRI: Kovats Retention Indices; **MW: molecular weight.

### Mastic oil inhibits colon cancer cell proliferation *in vitro* more effectively than its major constituents

**Figure 3:**
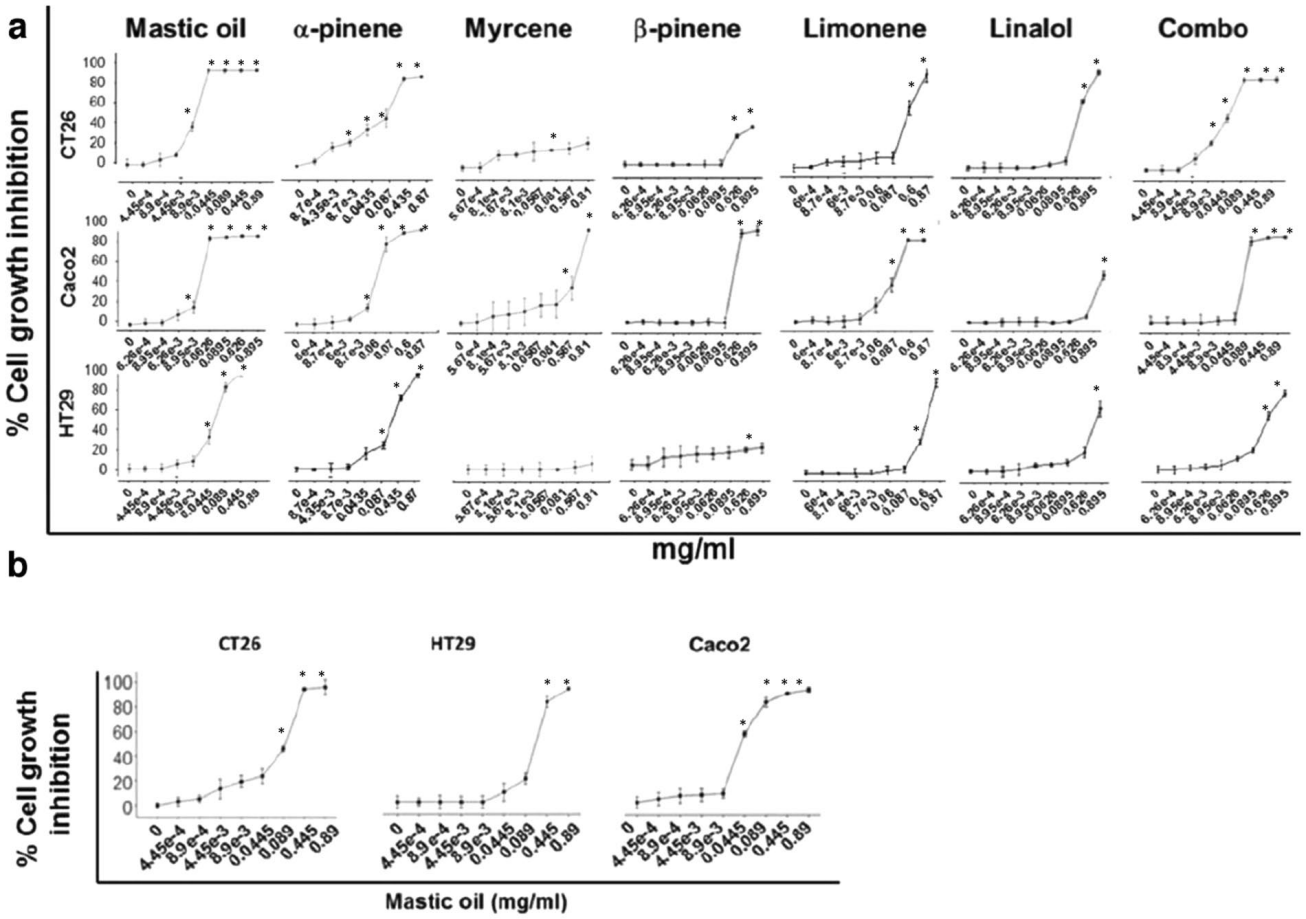
MO inhibits colon cancer cell proliferation *in vitro* more effectively than its major constituents. Antiproliferative effect of increasing doses of MO and its main constituents at (**a**) 72 h or (b) 48 h (only for MO) on murine CT26 and human HT29 and Caco-2 colon cancer cells, determined by the SRB assay. All data shown are representative of at least 3 independent experiments. Values represent mean (n = 6) ± SD.

MO and the monoterpenes α-pinene, β-pinene, myrcene, limonene and linalol were examined for their antiproliferative activity against human and murine colon cancer cell lines. MO inhibited growth of human and murine cells *in vitro*, in a concentration and time-dependent manner (Table 2, Fig. 3a,b). In addition, cytotoxic activity of MO and its monoterpenes was evidenced using the Trypan Blue exclusion test and by flow cytometry with propidium iodide (data not shown).

**Table 2:** IC_50_ values (efficient concentration that causes a 50% decrease in cell viability) of mastic oil (48 h and 72 h) and its constituents (72 h) against colon cancer cell lines. Data are representative of at least three independent experiments and are presented as mean ± SD (n = 6).

Cell line	Mastic oil IC_50_, 48 h (mg/ml)	Mastic oil IC_50_, 72 h (mg/ml)	α-pinene IC_50_, 72 h (mg/ml)	Myrcene IC_50_, 72 h (mg/ml)	β-pinene IC_50_, 72 h (mg/ml)	Limonene IC_50_, 72 h (mg/ml)	Linalol IC_50_, 72 h (mg/ml)	Combo IC_50_, 72 h (mg/ml)
CT26	0.1335 ± 0.0540	0.0104 ± 0.0004	0.2433 ± 0.0835	n.d.*	*n.d*.	0.4915 ± 0.0425	0.1540 ± 0.0267	0.0251 ± 0.0077
Caco-2	0.0368 ± 0.0225	0.0176 ± 0.0035	0.0720 ± 0.0012	0.6300 ± 0.0150	0.3700 ± 0.0701	0.0901 ± 0.0042	*n.d*.	0.0760 ± 0.0065
HT29	0.1751 ± 0.0028	0.0762 ± 0.0057	0.4837 ± 0.1211	*n.d*.	*n.d*.	0.6966 ± 0.0122	0.8428 ± 0.0126	0.4600 ± 0.0335

^*^ *n.d.*: not detected (not possible to determine efficient concentration that causes 50% decrease in cell viability).

The different cell lines exhibited different sensitivity to MO or its constituents. As expected, for a 72 hours incubation period, lower concentrations of the essential oil were needed to cause a 50% decrease in cell viability than for 48 hours (Table 2). The IC_50_ values for HT29 were determined as 0.1751 and 0.0762 mg/ml after 48 h and 72 h of incubation with MO, respectively. Caco-2 cells were more sensitive to the action of MO, with IC_50_ values of 0.0368 and 0.0176 mg/ml for 48 h and 72 h of incubation, respectively. Murine CT26 cells, showed lower sensitivity for 48 h of incubation (IC_50_: 0.1335 mg/ml) and comparable sensitivity to Caco-2 for a 72 h-treatment with MO (IC_50_: 0.0104 mg/ml). These data show that CT26 and Caco-2 are more sensitive to MO than HT29 cells. The major constituent in MO, α-pinene, also inhibited colon cancer cell proliferation, although to a lesser extent (IC_50_ after 72 h against HT29, Caco-2 and CT26: 0.4837, 0.0720 and 0.2433 mg/ml, respectively). Interestingly, the antiproliferative effect of α-pinene was enhanced upon combination with myrcene (referred as combo: ratio α-pinene/myrcene: 3.5/1w/v) in CT26 and HT29 cells (IC_50_ after 72 h: for HT29 0.4600 mg/ml, and for CT26 0.0251 mg/ml), despite the fact that myrcene alone did not exhibit a significant inhibitory effect. However, myrcene did not enhance the activity of α-pinene in Caco-2 cells, as in these cells, the combination of α-pinene and myrcene (combo) had similar antiproliferative activity to α-pinene (IC_50_ after 72 h 0.076 mg/ml for combo and 0.072 mg/ml for α-pinene) (Fig. 3, Table 2).

The strongest antiproliferative effect was induced by MO, to a lesser extent by the combination of α-pinene and myrcene or α-pinene. Much lower antiproliferative effect was exerted by the other monoterpenes: β-pinene, limonene, linalol and myrcene. Myrcene and β-pinene did not significantly inhibit growth of colon cancer CT26 and HT29 cells in the concentrations tested, nor did linalol in Caco-2 cells.

The strong antiproliferative activity induced by MO *in vitro* can be attributed to some extent to α-pinene; however, colon cancer cells were more sensitive to MO than to its individual monoterpenes, suggesting that, under these experimental conditions, on HT29, Caco-2 and CT26 colon cancer cells, there might be a potential synergistic effect among monoterpenes with the major constituent α-pinene.

### Combined cytotoxic effects of α-pinene and myrcene on Caco-2 cells

**Figure 4:**
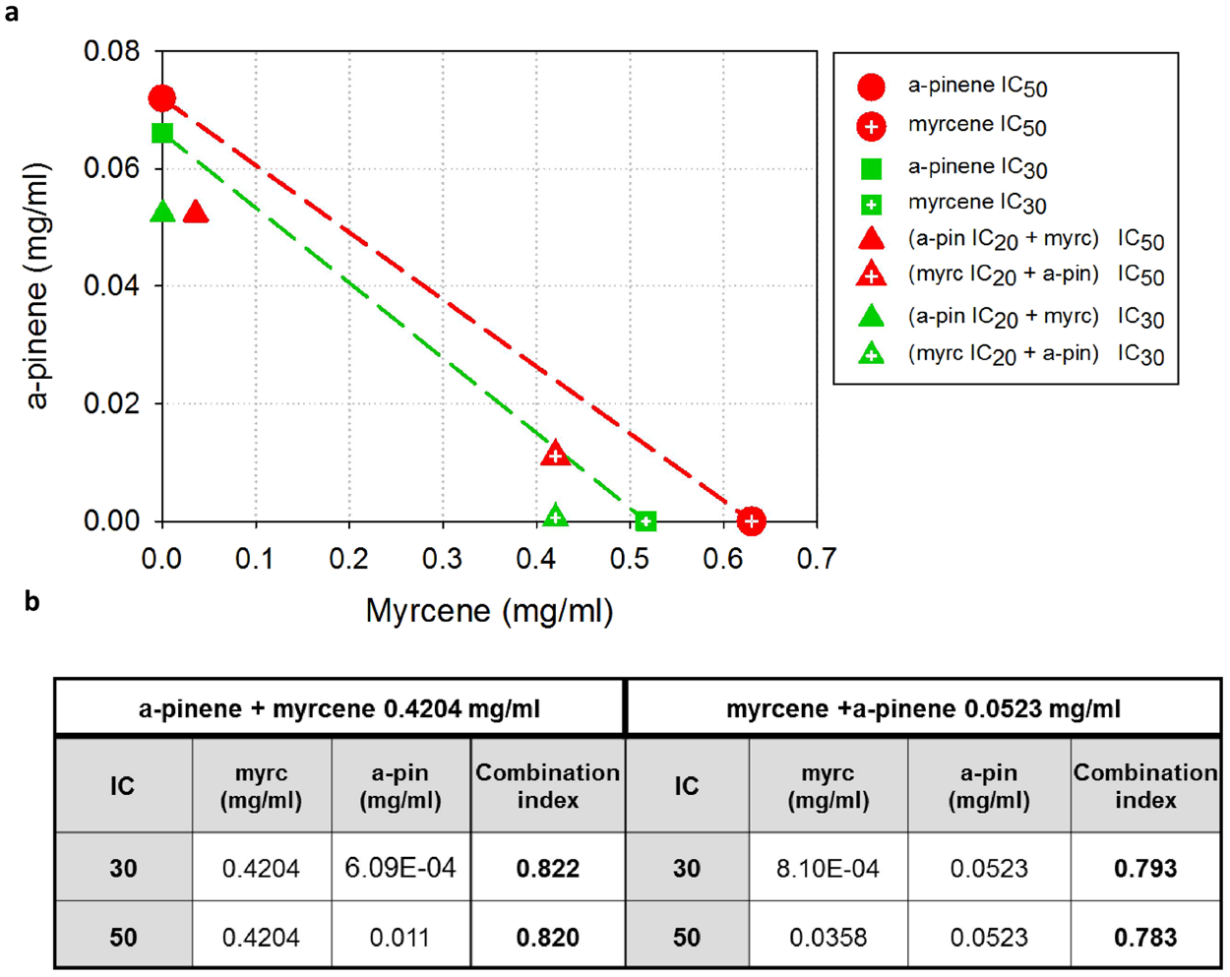
Synergistic Caco-2 cell growth inhibition by the combination of α-pinene and myrcene. (**a**) Isobologram showing the interaction between α-pinene and myrcene in inhibiting cell growth of Caco-2 cells. Cells were treated for 72 h with α-pinene and/or myrcene and their viability was estimated with the SRB assay. Red symbols denote the IC_50_ values of α-pinene (circle), myrcene (crossed circle), the combination of α-pinene’s IC_20_ and myrcene (triangle), and the combination of myrcene’s IC_20_ and α-pinene (crossed triangle). Green symbols denote the IC_30_ values of α-pinene (square), myrcene (crossed square), the combination of α-pinene’s IC_20_ and myrcene (triangle), and the combination of myrcene’s IC_20_ and α-pinene (crossed triangle). Dashed lines indicate additive effects. Solid data points below the line of the same color, indicate a synergistic effect, whereas points above the line, indicate antagonism. (**b**) IC_30_ and IC_50_ values of the different combinations of myrcene and α-pinene, and the estimated combination index values.

To evaluate the synergistic potential of the two major constituents of MO, α-pinene and myrcene, in cancer cell growth inhibition, we employed isobolographic analysis. Since the IC_50_ value of myrcene could be determined only on Caco-2 cells (Table 2), we selected this cell line for the isobolographic analysis. Moreover, because of the relevant low cytotoxic activity of myrcene, we analyzed α-pinene’s and myrcene’s synergism at low effect levels. Thus, cytotoxic interactions of α-pinene with myrcene at the 30% and 50% inhibitory concentration (IC_30_, IC_50_) levels were evaluated by the isobolographic method on Caco-2 cells. We used the IC_20_, IC_30_ and IC_50_ values of α-pinene and myrcene for Caco-2 cells as determined by the SRB assay and described in Fig. 3. We observed that the combination, enhanced their individual cytotoxic potential, since all the combination data points lie below the respective dashed lines in the isobologram (Fig. 4a) and the values of the calculated combination index for the 30% and 50% cell growth inhibition, are < 1 (Fig. 4b), indicating potential synergy. Details on the isobolographic analysis and the calculation of CI, can be found in the “Methods” section.

### Mastic oil attenuates migration of colon cancer *in vitro*

**Figure 5:**
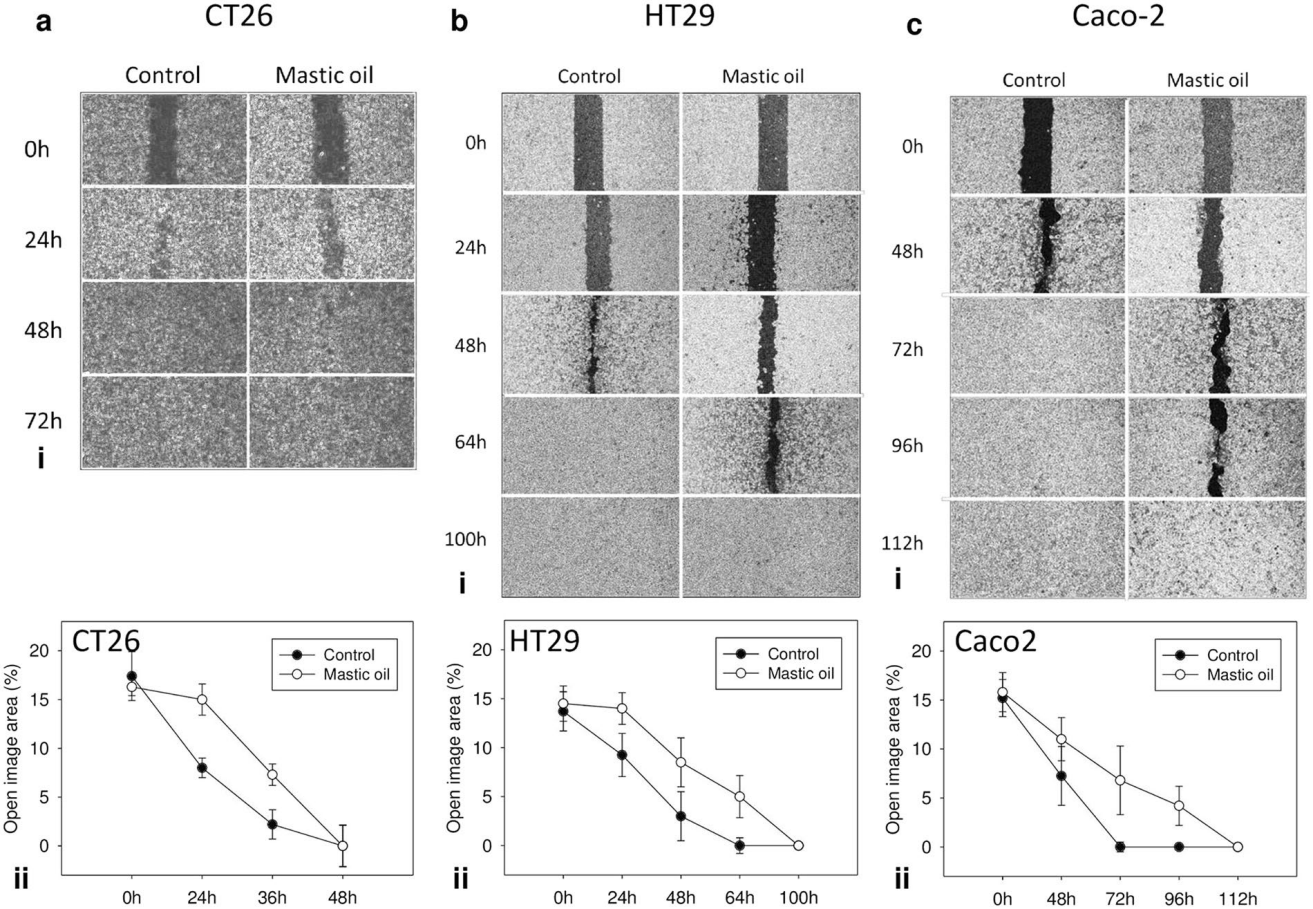
Effect of MO on migration of colon cancer cells. Wound-healing assay for (ai) CT26, (bi) HT29 and (ci) Caco-2 cells treated with MO (0.015 mg/ml for CT26, 0.02 mg/ml for HT29 and 0.004 mg/ml for Caco-2) or dimethylsulfoxide (DMSO) for control. Migration of cells was monitored with an optical microscope at the indicated time points. Quantification of the percentage of wound closure by ImageJ software analysis for (aii) CT26, (bii) HT29 and (cii) Caco-2 cells. Data are presented as the mean ± SD of three independent experiments.

Increased migration potential of cancer cells underlies tumor invasion^25^. In this context, we investigated the potential effect of MO on colon cancer cell migration rate *in vitro*. Wound healing assay on CT26, HT29 and Caco-2 cells confirmed that the open area (wound) closed earlier in control, DMSO-treated, cells compared to those that were treated with low, non-toxic concentrations of MO. Wound closure occurred after 48 h in MO-treated CT26 cells compared to 36 h in control, after 100 h in MO-treated HT29 cells compared to 64 h in control, and after 112 h in MO-treated Caco-2 cells compared to 72 h in control cells (Fig. 5). These results demonstrate that MO attenuates migration of murine CT26 and human HT29 and Caco-2 colon cancer cells, *in vitro*.

### Oral administration of Mastic oil inhibits *in vivo* growth of colon carcinoma in mice

**Figure 6:**
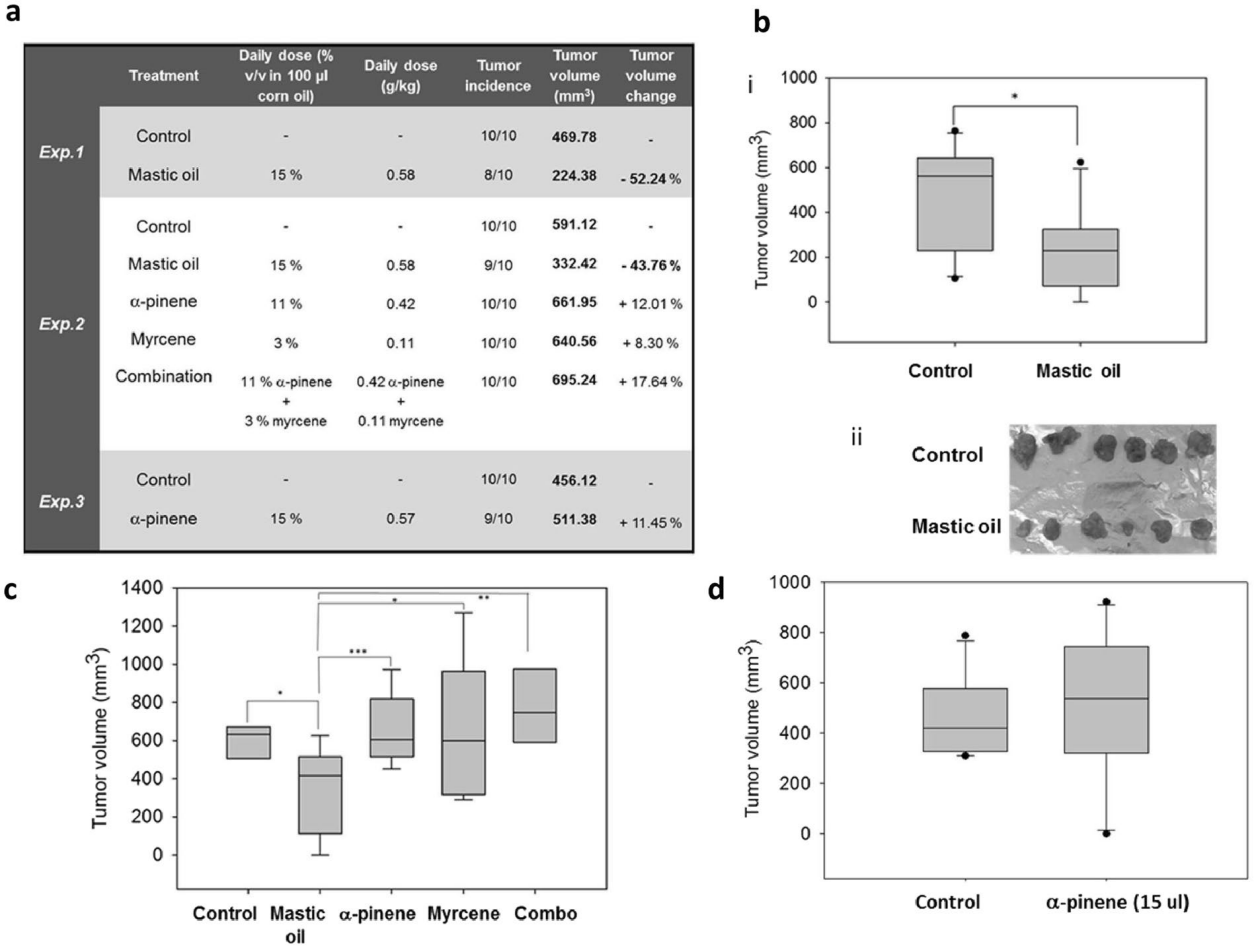
Oral administration of MO inhibits *in vivo* growth of colon carcinoma in mice. MO or α-pinene, myrcene or a combination of α-pinene and myrcene (combo) were administered *per os* daily to BALB/c mice for 13 days. On the tenth day mice were inoculated subcutaneously with CT26 cancer cells and 7 days later tumors were harvested from euthanized animals. (**a**) Presentation of results from three independent experiments (n = 10 per group) following oral administration of MO or its major monoterpenes. A statistically significant reduction of ≈43–52% in tumor volume (Exp.1: *p* = 0.017, Student’s *t*-test, Exp.2: *p* = 0.016, (one-way ANOVA)) was observed only in MO- treated mice as compared to control. (**b**) Mean tumor volume (bi) or photographic observation (bii) of tumors excised from mice that received MO or corn oil (control) (Exp.1). (**c**) Mean tumor volume of tumors excised from MO- or α-pinene (11% v/v)- or myrcene- or combo (a combination of α-pinene and myrcene)- treated mice (Exp.2) (**d**) Mean tumor volume from tumor bearing mice treated daily with a higher concentration (15% v/v) of α-pinene (Exp.3).

Oral administration of MO (0.58 g/kg body weight/day) for 13 days significantly inhibited tumor growth in mice compared to control animals, in two independent experiments, at a rate of 52% (*p* = 0.017) and 44% (*p* = 0.016), respectively (Fig. 6a–c). Tumors from MO-treated mice had a statistically significant lower tumor volume than tumors from control mice. Notably, α-pinene, the constituent with the most significant antiproliferative effect *in vitro*, did not induce tumor growth inhibition when administered orally, either alone in two dose-schemes (0.42 or 0.57 g/kg body weight/day) or in combination with myrcene at a dose equivalent to MO’s composition (0.42 g of α-pinene and 0.11 g of myrcene /kg body weight/day) (Fig. 6a,c,d). During the experimental procedure no signs of disease or discomfort were observed in all groups of mice.

Oral administration of MO but not its major constituents inhibits growth of colon carcinoma cells *in vivo* in an experimental CT26 colorectal tumor model. The *in vivo* data assume that tumor growth inhibition needs potential synergy among the monoterpenes present in MO.

### Mastic oil reduces protein expression of Ki-67 and survivin (BIRC5a) in colon cancer cells

**Figure 7:**
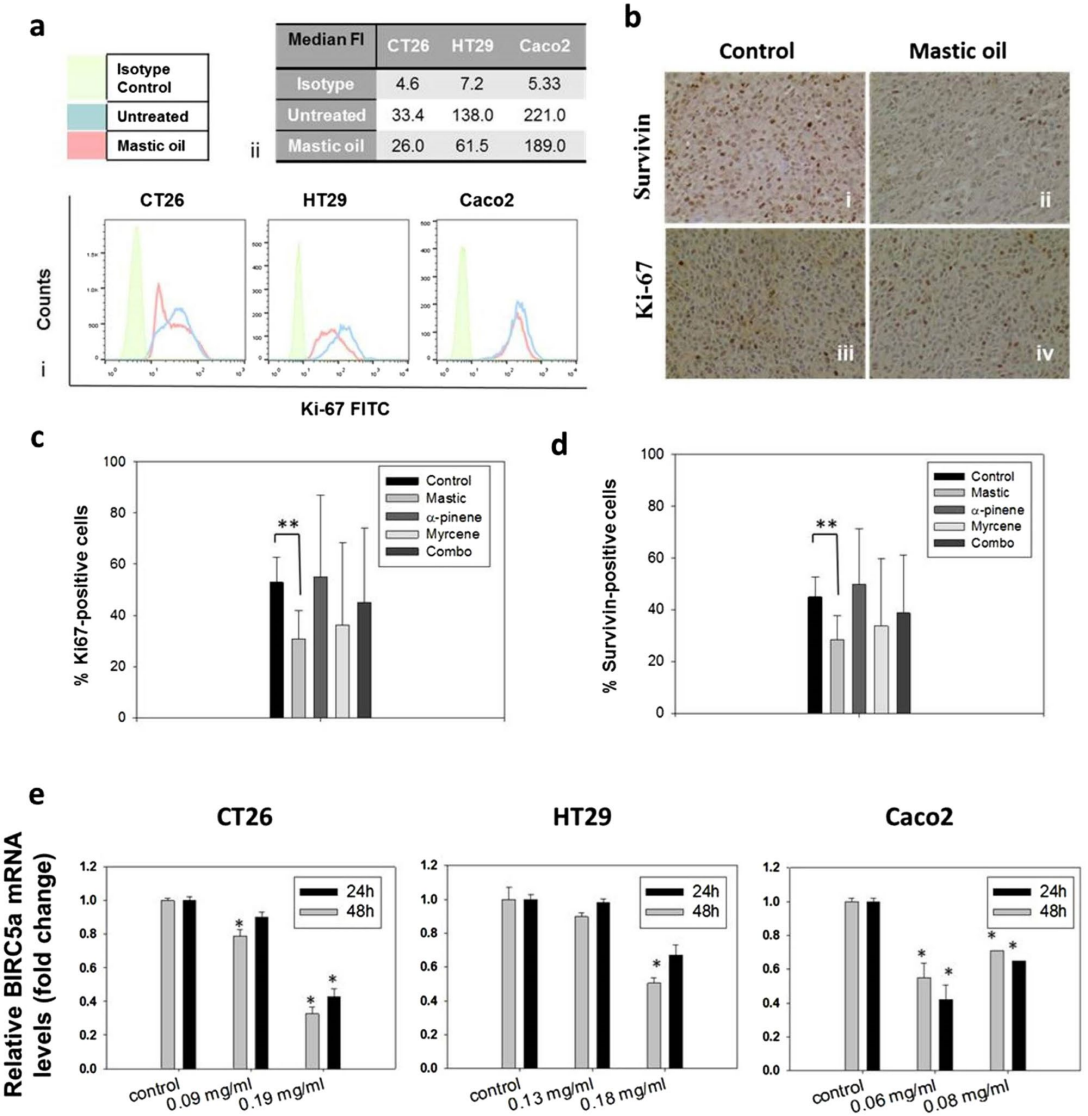
MO inhibits protein expression of Ki-67 and protein and transcriptional expression of survivin (BIRC5a). (**a**) Flow cytometric analysis of Ki-67 protein expression in MO-treated CT26, HT29 or Caco-2 colon cancer cell lines compared to non-treated cells. Results are representative of three independent experiments. (**b**–**d**) Immunohistochemical analysis on tumors excised from mice treated *per os* with MO or its major constituents. Representative images (**b**) showing the effect of administration of MO on survivin (bi,bii) and Ki-67 (biii,biv) protein expression. Results showing the percentage of Ki-67-positive (**c**) or survivin-positive (**d**) cells in CT26 tumors excised from BALB/c mice treated with MO or its constituents. Statistically significant differences were observed in (**c**) the number of Ki-67 positive (*p* = 0.003, Student’s *t*-test) or (**d**) the number of survivin-positive (*p* = 0.049, Student’s *t*-test) cells in tumor tissue from MO-treated mice as compared to control mice. Each bar represents the mean number of positive cells ± SD in tumor sections from at least three mice. (**e**) Relative gene expression (mean fold change) of *BIRC5α* in CT26, HT29 or Caco-2 cells treated with MO for 24 or 48 hours, as compared to non-treated cells. Mean fold change (±SD) is relative to control cells recovered before treatment. Endogenous expression of *ACTB* was used as internal reference. Results are representative of three independent experiments and are presented as mean values of triplicates ± SD. Asterisks indicate statistically significant differences (*p* < 0.05, Student’s *t*-test).

MO reduced the protein expression of the proliferation marker Ki-67 in three colon cancer cell lines *in vitro* (Fig. 7a). After treatment of HT-29 cells for 24 h with 0.178 mg/ml MO, the median fluorescence intensity for Ki-67 expression was reduced from 138 in control cells to 61.5. In accordance, a drop in protein expression of Ki-67 was also observed for Caco-2 cells. Median fluorescence intensity was reduced from 221 in control cells to 189 in cells treated with 0.180 mg/ml MO. For CT26 cells, median fluorescence intensity was reduced from 33.4 in control cells to 26.0 in cells treated with 0.250 mg/ml MO (Fig. 7a). In addition, a reduced expression of Ki-67 and survivin in tumor tissues accompanied the observed growth inhibitory effect of MO (Fig. 7b,c,d). A statistically significant reduced number of Ki-67- or survivin-expressing cells was observed in tumor tissues of mice treated orally with MO than control ones, as determined by immunohistochemical analysis using specific antibodies (Fig. 7b). The percentage of Ki-67 positive cells was 27 (±13.8%) compared to 53 (±9.9%) in control mice, *p* = 0.003), a reduced number of Ki-67 positive cells was also observed in myrcene- or combo-treated mice (Fig. 7c). Furthermore, survivin-mRNA levels were downregulated in a concentration and time-dependent manner in HT29, Caco-2 and CT26 treated with MO (Fig. 7e). Moreover, a reduced expression of survivin in CT26-tumors was observed after oral administration of MO (Fig. 7b). The percentage of survivin-positive cells was 28% (±9.3) in MO-treated mice compared to 45% (±7.6) in control mice (*p* = 0.005). Survivin expression was also inhibited in myrcene- and combo-treated mice but not in a statistically significant manner (Fig. 7d).

## Discussion

During the last decade the health promoting benefits of the oil and other extracts derived from Chios mastic resin attracted considerable interest, due to the antimicrobial, anti-inflammatory, anti-oxidant and other biological properties, as well as the anti-cancer potential of MO^16, 24, 26–30^. In the present study, we focused on the dietary benefits of MO in colon cancer and evaluated MO’s biological activities in an experimental gastrointestinal-relevant neoplasia model. In particular, we have shown that colon cancer cells are sensitive to the activity of MO or its major constituents. In three colon cancer cell lines the antiproliferative effect of MO was more potent than the growth-inhibitory effect induced by the individual monoterpenes (α-pinene, myrcene, β-pinene, limonene or linalol) present in MO. The most significant growth inhibitory effect, besides MO, was exerted by the major constituent α-pinene, and to a much lesser extent by the other monoterpenes. Moreover, MO inhibited the growth of colon carcinoma tumors *in vivo* in mice following daily oral administration, indicating that oral administration of MO has antitumor potential in a transplantable mouse tumor model of colon cancer. We also provide evidence that MO’s antiproliferative potency against colon cancer cannot be attributed on one of its main components, implying the presence of synergistic or additive interactions between MO’s constituents.

The composition of MO extracted from the mastic resin was analyzed by GC/MS, and α-pinene (68%) and myrcene (19%) were identified as MO’s major constituents. Our results are in accordance with literature data showing that MO is mainly a mixture of α-pinene (38-80%) and myrcene (3-20%)^29, 30^. The monoterpenes β-pinene (3.0%), limonene (0.9%) and linalol (0.73%) were also identified among the most abundant phytochemicals in MO following α-pinene and myrcene.

We have shown that MO inhibited cell growth of murine (CT26) and human (Caco-2 and HT29) colorectal cancer cells *in vitro*. The lowest IC_50_ values (72 h) of MO compared to its main constituents in all examined cell lines indicate that the observed growth inhibitory effect of MO is a result of the combined activities of more than one of its constituents. Evidence on the synergistic anticancer effects of MO’s components have been previously proposed^21^, but not comparatively examined. After treating all cell lines with MO or its five most abundant individual constituents, we concluded that the strongest antiproliferative effect was induced by the combination of all terpenes present in MO, and to a much lesser extent by α-pinene, with an IC_50_ value for α-pinene, four (Caco-2) to twenty-three (CT26) times higher than for MO. The other monoterpenes β-pinene, limonene and linalol showed much lower antiproliferative effect while myrcene did not significantly inhibit growth of colon cancer cells at the concentrations tested. To our knowledge, this is the first report comparing the growth-inhibitory activity of MO with each one of the five major monoterpenes present in MO. Since MO is a mixture of volatile terpenes, the outcome we observed is due to the combined action of all these compounds, taking into consideration that each individual terpene may target different intracellular signaling pathways.

Although the sensitivity was different among the mouse and human cell lines, the trend was similar in all cell lines tested. Notably, both human cell lines are suitable models for *in vitro* study of the functional properties of bioactive compounds; Caco-2 cells can be regarded as a model of small intestine, whereas HT-29 cells, as a model of large intestine^31^. Moreover, these cell lines have different genetic and epigenetic alterations and different mutations in p53^32^. Differences in susceptibility to phytochemicals have also been reported^33^ as well as differences in the expression of cell death-related proteins. For example, the triterpene maslinic acid is capable of inducing apoptosis via both the intrinsic and extrinsic apoptotic pathways, depending upon the type of colon cancer cells involved. Maslinic acid triggers the extrinsic mechanism for apoptosis on Caco-2 colon-cancer cells^34^ as opposed to the intrinsic mechanism in HT29 colon cancer cells^35^. Therefore, we believe that the differences in the IC_50_ among the different cell lines may be attributed to different molecular signaling pathways that are targeted in each particular cell line by the combined action of the constituents present in MO.

The high levels of α-pinene and myrcene in MO and the relatively strong (compared to constituents) inhibitory effect of α-pinene in contrast to myrcene, made us speculate whether there might be a synergistic or additive effect among α-pinene and myrcene. Treatment of CT26, Caco-2 and HT29 cancer cells with a mixture-combination of α-pinene and myrcene in a v/v rate equivalent to MO’s constitution (3.5:1 w/v), enhanced α-pinene’s antiproliferative activity in CT26 and HT29 cells, but did not exceed the growth-inhibitory effect of MO in all cell lines tested. To further evaluate the combined effect of α-pinene and myrcene, we analyzed their cytotoxic interactions on Caco-2 cells with combination index and isobologram analysis. The *in vitro* experimental validation, revealed a synergistic effect between α-pinene and myrcene depicted by the CI_30_ and CI_50_ values, that were all calculated to be < 1 (Fig. 4b). By this evidence, we assume that MO’s *in vitro* anticancer activity against colon cancer cell lines may be attributed to the combined activity of more than two of its main constituents, even though, the latter do not inhibit cell growth when tested individually. In addition to cell growth, MO affected the migratory properties of CT26, Caco-2 and HT29 cells as it was evidenced by the wound healing assay. MO treatment with sub-toxic concentrations, significantly inhibited cell migration in all colon cancer cell lines tested.

Although reported observations showed a strong direct antitumor effect of MO when administered intraperitoneally in a mouse lung carcinoma model^21^, the potential antiproliferative activity of orally administrated MO has not been previously studied. MO is a flavoring agent that is currently being used in the food industry (in the bakery and confectionery products, in liqueur and soft drinks, etc.). Moreover, its alleviating effects on gastrointestinal disorder symptoms have been widely known and thus, MO has been commonly used in traditional medicine. Furthermore, the resin from *Pistacia lentiscus* var. *chia* has been recently certified as a natural medicine by the European Medicines Agency^36^ but the mechanisms for its beneficial effects are not still understood. On these grounds, it was of particular importance to investigate whether dietary MO has any beneficiary prophylactic effect against colon cancer.

We found that MO inhibited *in vivo* growth of colon carcinoma tumors in mice following daily oral administration with a tumor volume inhibition of 44–52%. Interestingly, α-pinene, MO’s main constituent and the compound that showed the greatest antiproliferative effect *in vitro* (after MO), did not show antitumor effect under the same *in vivo* experimental design nor did α-pinene’s mixture with myrcene, a combination that was proven to enhance α-pinene’s *in vitro* antiproliferative effect against CT26 cells. In accordance with the *in vitro* results, our data suggest combined antitumor effects induced by MO’s constituents. Along similar lines, there is a great number of recent reports arguing that naturally occurring combinations of phytochemicals, possess enhanced biological reactivity^37–39^.

Moreover, we investigated MO’s and its major constituents’ effect on key proteins involved in cell proliferation and tumor growth. A reduced protein expression of the proliferation marker Ki-67 as well as reduced mRNA expression of survivin (a tumor progression marker) was observed in all colon cancer cell lines *in vitro* following treatment with MO compared to control non-treated cells. In addition, a reduced expression of Ki-67 and survivin in tumor tissues from mice treated orally with MO, accompanied the observed tumor-growth inhibitory effect. In recent years, there has been an increasing interest in targeting survivin expression to develop novel therapeutic approaches for cancer. Survivin holds a prominent role in both cell division and apoptosis, two crucial processes in cancer development. Moreover, survivin can induce an effective CTL response^40^. Ki-67, besides being a cellular marker for proliferation, it has been used for cancer prognosis and has been recently proposed to also be an attractive potential therapeutic target for numerous malignancies^41^. Notably, in contrast with MO, a lower percentage of Ki-67- and survivin-expressing cells in tumors excised form mice treated with myrcene or combo, did not associate with inhibition of tumor growth, supporting the significance of MO’s phytochemical’s synergy in inhibiting tumor growth.

Interestingly, the MO concentrations used in our experiments can be regarded as safe, since there was no toxicity observed in the short-term oral administration of MO in mice, in accordance to the findings of another study examining the effect of MO in different tissues^42^. MO did not considerably alter the redox or detoxification mechanisms in different tissues^42^. Noteworthy, MO or mastic water extract were previously shown to lack genotoxic or mutagenic activities^43, 44^. Many studies also demonstrated lack of genotoxicity for β-myrcene or α-pinene in *in vitro* and *in vivo* systems^45–48^ however, in one study α-pinene was shown to compromise genomic instability^49^. Notably, various monoterpenes such as limonene and perillic acid have been reported to possess antitumor activity^10^. The lack of genotoxicity in combination with the cytotoxic activity exhibited by MO and many of its constituents is suggestive of a natural non-toxic product with pharmacologic potential in anticancer medicinal treatments.

In conclusion, our work provides novel evidence that oral administration of MO, an oil used as flavoring agent in food industry, exerts anticancer activities by attenuating tumor growth of colorectal cancer in a mouse tumor model. The observed antitumor effect could be attributed to combined activities of MO’s phytochemical terpenes. Our findings suggest that there might be a great potential in the use of MO as a beneficial dietary neutraceutical for colon cancer prevention. Moreover, these results are of particular value in the characterization of the anticancer activity of MO, and set the stage for subsequent evaluation of MO’s neutraceutical potential in clinical studies. Further research on the underlying molecular mechanisms is of utmost importance in order to identify cellular target pathways where MO and its constituents exert their anti-tumor efficacy.

## Materials and Methods

### Essential oil, monoterpenes, and reagents

Mastic gum was kindly provided by Chios Mastic Gum Growers Association L.L.C. (Chios, Greece). The air dried resinous gum is collected by hand from the plant or from its surrounding area. The MO was produced with the use of a small experimental distillation equipment under vacuum in VIORYL’s research laboratories. Mastic gum was slightly milled and directly heated (50-110 °C). The distillation process resulted in some viscous fractions where the unresolved matter was removed by centrifuging (1,000 g for 10 min) the sample and then separating the upper layer (essential oil) with a syringe. The total distillate was used without any further fractionation. The monoterpene compounds α-pinene 90-93% (TREATT), β-pinene 97% (LLUCH), myrcene 91-93% (TAKASAGO), limonene 99% (VIORYL) and linalol 98% (BASF) were used. All other chemicals were purchased from Sigma-Aldrich.

### Gas chromatography and mass spectroscopic analysis (GC/MS)

GC/MS analysis of MO was performed out in a GC-MS (GC: 6890 A, Agilent Technologies, USA; MSD: 5973, Agilent Technologies) using a Factor Four VF 1ms column (25 m, 0.2 mm i.d., 0.33 μm film thickness, Agilent Technologies). 0.1 μl of essential oil was directly injected and a 1:100 split ratio was applied. The oven temperature was set at 50 °C for 1 min, followed by a temperature gradient of 2.5 °C/min. When temperature reached 160 °C it was kept steady for 20 min. Then a step of 50 °C/min was applied until oven temperature was 250 °C, where it was kept for 15 min. Helium was used as carrier gas with a flow rate of 1 ml/min. Injector and transfer line temperatures were set to 200 °C and 250 °C, respectively. The mass spectrometer operated in the electron impact mode with the electron energy set to 70 eV. Volatiles identification was completed according to the standard method of Kováts Indices and mass spectra comparison to Willey/NIST 0.5 and in-house created libraries (VIORYL S.A.).

### Cell lines

Human HT29 and Caco-2 and murine CT26 colon carcinoma cell lines were maintained under sterile conditions at 37 °C in a humidified atmosphere of 5% CO_2_, and routinely cultured in DMEM (HT29 and CT26) or RPMI-1640 (Caco-2) medium, both supplemented with 10% fetal bovine serum (Biosera), penicillin (100 U/mL) and streptomycin (100 μg/mL) (Biosera), and 2 mM Glutamine (Gibco).

### Sulforhodamine B (SRB) Assay

Cell viability was determined by the SRB assay^50^. Briefly, cells were seeded in 96-well plates at an initial cell density of 5,000, 20,000 or 4,000 cells per well for CT26, HT29 and Caco-2 cells, respectively. Cells were treated with increasing concentrations of MO or its major monoterpenes dissolved in DMSO (1:1 v/v) for 48 h or 72 h. Control cells were incubated in DMSO-containing DMEM (DMSO concentration ≤ 0.1% v/v). Then, cells were fixed with 10% TCA at 4 °C for 1 h, and dried. Cells were stained with SRB (0.057% w/v) for 30 minutes at room temperature. After staining, cells were repeatedly washed with 1% acetic acid and left to dry. The dye was dissolved in 10 mM Tris base, and absorbance was measured at 492 nm using a microplate reader (Enspire, Perkin Elmer). The IC_50_ values (efficient concentration that causes a 50% decrease in cell viability) were calculated from the respective dose response curves by regression analysis using the Sigma Plot Software (v.11).

At least five replicates for each sample were examined and each experiment was independently performed at least three times. The % inhibition of cell growth was calculated by the following formula:

1 $$ \% growth\,inhibition=100-(mean\,OD\,sample/mean\,OD\,control\times 100)$$

### Isobolographic analysis of myrcene and α-pinene

The potential synergy of α-pinene and myrcene, regarding the inhibition of Caco-2 cell proliferation, was assessed using combination index (CI) and isobolographic analysis based on Lowe additivity^51^. The IC_20_ values for 72 h of α-pinene and myrcene on Caco-2 cells, are 0.0523 and 0.4204 mg/ml, respectively. The IC_30_ values are 0.0661 and 0.5171 and the IC_50_ values are 0.0720 and 0.6300 mg/ml for α-pinene and myrcene, respectively. IC values were determined by the results of the SRB assay (Fig. 3). Caco-2 cells were treated for 72 h with the IC_20_ of α-pinene combined with different concentrations of myrcene and vice versa. Viability of cells was assayed with the SRB method and data were analyzed with SigmaPlot v11. For each series of combinations, the IC_50_ and IC_30_ values were determined and plotted along with the IC_30_ and IC_50_ values of α-pinene and myrcene (Fig. 4). The theoretical additive effect of the two compounds is depicted by the dashed lines that connect the two points, representing the iso-effective concentrations of α-pinene and myrcene (either of red color for IC_50_, or green for IC_30_). If the experimentally estimated IC_50_ and IC_30_ values of the different combinations of the examined compounds are plotted by data points that lie below the respective dashed line, we can assume that the compounds act synergistically. On the other hand, if the data points lie above the dashed line, the two compounds are antagonistic. Moreover, we determined α-pinene’s and myrcene’s combination index (CI) whose value indicates the degree of synergism or antagonism between two compounds. More specifically CI < 1, = 1, or > 1 indicates synergistic, additive or antagonistic effect, respectively^52^.

CI was calculated using the equation:

2 $$C{I}_{x}={C}_{1,x}/I{C}_{x,1}+{C}_{2,x}/I{C}_{x,2}$$

where CI_x_ stands for the combination index based on the effect of x% cell growth inhibition (either 50% or 30% here), C_1,x_ and C_2,x_ represent the concentrations of compounds 1 and 2 (α-pinene and myrcene), used in combination for inducing the same x% inhibition, and IC_x,1_ and IC_x,2_ represent the iso-effective concentrations of the same compounds that, when used individually, induce the same x% cell growth inhibition as their combination^52^. Representative results of at least three independent experiments are being presented.

### Wound healing assay

CT26, Caco-2 and HT29 cells were seeded in 35-mm culture dishes with IBIDI silicon inserts (IBIDI GmbH) consisting of two reservoirs separated by a 500 μm wall. 3 × 10^5^ cells/ml were seeded in 70 μl of standard DMEM culture medium per reservoir. One insert was used per dish, and two dishes were seeded per cell line. After an overnight incubation at 37 °C and 5% CO_2_, the IBIDI insert was removed creating a 500 μm wide wound. In order to exclude the possibility that the wound healing process is attenuated due to the growth inhibitory effects of MO, we used non-toxic concentrations for the treatment of the cells that did not inhibit cell growth. Cells were treated with MO (0.015 mg/ml for CT26, 0.020 mg/ml for HT29 and 0.004 mg/ml for Caco-2) or DMSO (control, DMSO concentration ≤ 0.1% v/v) and photographed at indicated time points with a ZEISS Primovert light microscope (Zeiss, Göttingen, Germany) equipped with a digital camera (Axiocam ERc 5 s). Multiple photographs per time point were analyzed with ImageJ software (NIH, USA) and the average % wound area (% open image area) was calculated.

### Flow cytometric analysis of Ki-67 expression

For the flow cytometric analysis of Ki-67 expression in colon cancer cells, FITC Mouse Anti-Human Ki-67 Set (BD Pharmigen) was used according to the manufacturer’s protocol. Briefly, 3 × 10^5^ cells/well were seeded in 6-well plates. Following overnight incubation, cells were incubated with different concentrations of MO or DMSO–containing DMEM (max. DMSO concentration: 0.02% v/v) for 24 h. Cells were trypsinized, fixed in ice-cold 70% ethanol and stored at −20 °C overnight. Before the flow cytometric analysis, cells were washed twice with PBS containing 1% FBS, resuspended adjusting at a final concentration of 1 × 10^7^ cells/ml in the same buffer, and stained with Ki-67 antibody or Isotype control for 30 min. Cells were washed and analyzed on a flow cytometer (Calibur, BD Biosciences) for the detection of Ki67-FITC (FL1) intensity. Cell debris and dead cells were excluded from the analysis based on scatter signal.

### Animals and CT26 experimental tumor model

Female BALB/c mice (6–8 weeks old, weight 20–25 g) were purchased from the Animal Facility of Pasteur Institute (Athens, Greece) and kept in the Animal House of Medical School at the University of Ioannina (Greece). Mice were housed in polycarbonate cages, max. 10 mice per cage, at room temperature, on a 12 h light-12 h dark cycle and were provided with tap water *ad libitum* and a commercial pelleted diet (Mucedola). The experimental protocol was approved by the Animal Care and Use Committee of the Veterinary Service in Ioannina and was in compliance with Directive 86/609/EEC. Female BALB/c mice were separated into independent groups (10 mice per group). A total of 90 female mice in three independent experiments were used. For 13 days, MO, α-pinene, myrcene and a mixture of α-pinene and myrcene (combo), that was proven to enhance α-pinene’s *in vitro* antiproliferative effect, were administered *per os* in a final volume of 100 μl, at a daily dose of 0.58, 0.57 or 0.42, 0.11 and 0.42 + 0.11 g/kg of animal body weight, respectively. Mice in the control group received an equal volume of corn oil (vehicle). At day 10, 5 × 10^6^ CT26 cells per mouse were injected subcutaneously as a single dose, and seven days post CT26 inoculation, mice were euthanized by cervical dislocation and tumors were excised. Tumor volume and incidence were determined. Tumor dimensions were measured by an electronic micrometer and tumor volume was calculated using the modified ellipsoid formula [(width^2^ × length)/2]. During the course of the experiments the weight change of each mouse was recorded and all mice were monitored for signs of disease or discomfort.

### Immunohistochemical Analysis

Tumors were fixed in 10% formalin (Merck) and then dehydrated in graded concentrations of ethanol, xylole (Diapath) and finally embedded in paraffin (Diapath). Serial sections 3 μm thick were prepared from the formalin-fixed, paraffin-embedded tissue blocks and floated onto charged glass slides. A hematoxylin (Merck) and eosin (Diapath) stained section was obtained from each tissue block. Immunostaining was performed on formalin-fixed, paraffin-embedded tissue sections by the streptavidin-biotin peroxidase labeled method. All sections were deparaffinized and hydrated using graded concentrations of ethanol to deionized water. Tissue sections were subjected to quenching of endogenous peroxidase and antigen retrieval using microwaving in low pH citrate buffer (pH 6). Primary antibodies, anti-Ki-67 (Cell Signaling CST, dilution 1:50) or anti-survivin (Cell Signaling CST, dilution 1:50), were then applied to the tissues and incubated overnight at 4 °C). Bound antibody was then visualized with DAB chromogen (Dako), followed by counterstaining with hematoxylin. Tissue sections incubated only with secondary antibody served as negative controls. An image analysis system composed of the Olympus BX43 upright microscope, digital camera Olympus Cam-SC30 and soft analysis (analySISH) was used in the tumor sections (stained with antibodies and counterstained with hematoxylin). The immunohistochemical expression of Ki-67 or survivin was nuclear (Fig. 7b). A continuous score system was adopted by using the x40 objective lens and counting at least 10 fields selected on the basis that they contained immunopositive tumor cells. The number of immunopositive cells was divided by the total number of the counted cells, and the expression was defined as the percentage of positive cells in the total number of the counted cells. The scoring was performed by evaluation of staining by two observers using light microscope. Tumor sections from at least four animals per group were analyzed.

### RNA extraction, cDNA synthesis, and Real-time PCR analysis

To analyze *BIRC5α* gene expression in colon cancer cells treated with MO, CT26, HT29 and Caco-2 cells were seeded in 6-well plates at a density of 5 × 10^5^ cells/well. After an overnight incubation, cells were treated either with MO (0.09 or 0.19 mg/ml for CT26, 0.13 or 0.18 mg/ml for HT29 and 0.06 or 0.08 mg/ml for Caco-2 cells) or DMSO (control, DMSO concentration ≤ 0.1% v/v) for 24 h or 48 h. After the treatment period, total RNA was extracted from the cells, using the Trizol reagent (Invitrogen). Quality and concentration of RNA were examined by ethidium bromide-stained agarose gel electrophoresis and spectrophotometric analysis. One microgram of RNA was used for reverse transcription and synthesis of cDNA template with the PrimeScriptcDNA synthesis kit (Takara, Saint-Germain-en-Laye, France). Quantitative real-time PCR was performed on a StepOne PCR System (Applied Biosystems) in MicroAmp® Fast Optical 48-Well Reaction Plates or MicroAmp™ Optical 8-Cap Strips using the KAPA SYBR Fast MasterMix ABI Prism (KAPA Biosystems) reagent. The thermal cycling conditions were 95 °C for 2 minutes followed by 40 cycles of 95 °C for 2 seconds and 60 °C for 30 seconds. RT-PCR primers (VBC Biotech) were designed using Primer3 software to have the same Tm (60 °C) and were as follows; murine *BIRC5α* forward primer: GACCACCGCATCTC and reverse primer: AAGTCTGGCTCGTTC; murine *ACTB* forward primer: CGGTTCCGATGCCCTGAGGCTCTT and reverse primer: CGTCACACTTCATGATGGAATTGA, human *BIRC5a* forward primer for Caco-2 cells: ATCCACTGCCCCACTGAGAA and reverse primer: AGCTCCTTGAAGCAGAAGCAC; human *BIRC5a* forward primer for HT29 cells: CAAGGAGCTGGAAGGCTG and reverse primer: TTCTTGGCTCTTTCTCTGTCC; human *ACTB* forward primer: GCGCGGCTACAGCTTCA and reverse primer: CTTAATGTCACGCACGATTTCC. Primer specificity was verified by performing a melting curve analysis. Endogenous expression of *ACTB* (beta actin) was used as the internal reference. *BIRC5a* mRNA expression levels were evaluated by the comparative quantification Ct method (ΔΔCt)^53^. Statistical analysis was performed by SPSS 19 software. Normality was determined with the Kolmogorov-Smirnov test and groups were analyzed with a Student’s *t*-test.

### Data Analysis and Statistics

Data are presented as mean ± SD. Data were analyzed with statistical software (Sigma Plot v. 11.0 or SPSS 19). Normal distribution was examined using the Shapiro-Wilk test unless otherwise stated. Statistical comparisons between groups were performed using the Student’s *t-test* or one-way ANOVA were appropriate. Differences between control and treated groups were considered statistically significant when *p* < 0.05 (*p < 0.05, **p < 0.01, *** *p* < 0.001).

### Ethics statement

Animal experiments were approved by the Animal Care and Use Committee of the Veterinary Department of Ioannina Prefecture (license number EL20BIO02) since it complied with the requirements set by Directive 86/609/EEC and PD 160/91 which was the legislation in force at the time of experimentation. All animal experiments were conducted in light of 3 R’s (replacement, refinement, reduction) and all mice used for the experiments were not subjected to pain or discomfort.
